# Workplace Incivility, Bullying, and Mental Health-Related Outcomes Among University Faculty: A Narrative Review

**DOI:** 10.7759/cureus.104501

**Published:** 2026-03-01

**Authors:** Asako Matsuura

**Affiliations:** 1 Division of Translational Nursing, Toho University, Funabashi, JPN

**Keywords:** mental health, mobbing, narrative review, workplace bullying, workplace cyberbullying, workplace incivility

## Abstract

University faculty, particularly in health-related disciplines, perform multiple roles in teaching, research, administration, and student support. Consequently, negative interpersonal behavior may have meaningful implications for well-being and retention. Workplace incivility involves low-intensity disrespect with ambiguous intent; workplace bullying or mobbing involves repeated negative acts over time, often with a power imbalance; and workplace cyberbullying occurs via electronic communication.

We conducted a narrative review with a structured search and selection approach to summarize quantitative studies examining workplace incivility, workplace bullying/mobbing, and workplace cyberbullying among university faculty and their associations with mental health and work-related outcomes. The search used English-language terms in PubMed and Cumulative Index to Nursing and Allied Health Literature (CINAHL) and Japanese/English keyword combinations in Ichushi-Web.

Six studies met our inclusion criteria. Across studies, higher levels of incivility or uncivil workplace environments were associated with lower job satisfaction and higher turnover intentions; one two-wave study suggested negative work rumination as a mediating factor. Evidence on bullying and mobbing indicates associations with occupational stress symptoms and, in a clinical/forensic evaluation context, substantial psychiatric symptom burden. Evidence on workplace cyberbullying is limited but suggests a negative association with job satisfaction.

## Introduction and background

University faculty members in health-related disciplines frequently face overlapping job demands. Beyond classroom and clinical teaching, many are expected to publish, obtain funding, contribute to committees, and advise students in resource-constrained environments.

Among negative interpersonal behaviors, workplace incivility and bullying have received increasing attention. Incivility refers to low-intensity behavior that violates norms of mutual respect, with ambiguous intent to harm. Bullying (including mobbing) generally involves repeated negative acts over time and is often characterized by a power imbalance [1-7].

Higher education may provide conditions that facilitate these behaviors. Faculty work is embedded in evaluation processes tied to promotion, tenure, resource allocation, and informal influence networks. Ambiguous rules and hierarchical structures may allow small violations of respect to accumulate while discouraging reporting because of fear of retaliation or professional disadvantage. This may be particularly relevant for health-related faculty members who operate across academic and clinical settings, where multiprofessional relationships and role expectations can be complex [8,9]. A validation study in Japan also supported the reliability and validity of a Japanese incivility scale and reported associations with psychological distress and turnover intention [10].

Although this review is motivated in part by concerns about faculty well-being in health-related disciplines (e.g., nursing education), the evidence base in this area includes studies of university faculty more broadly. Therefore, this review synthesizes quantitative evidence on university faculty in general, while interpreting implications with particular attention to health-related faculty contexts.

The objective of this narrative review was to organize and interpret quantitative evidence on how workplace incivility, workplace bullying/mobbing, and workplace cyberbullying are associated with mental health and work-related outcomes among university faculty and to identify key conceptual and measurement issues relevant to future research and practice.

## Review

Methods

Design and Rationale

This study was conducted as a narrative review with a structured search and selection approach. The aim was to describe research trends and clarify conceptual, measurement, and interpretive issues rather than to provide pooled effect estimates. Accordingly, this review was not designed as a systematic review or a scoping review, and it does not claim exhaustive coverage or full reproducibility. To improve transparency and reduce arbitrariness, however, we prespecified eligibility criteria, documented the search approach, and used a fixed data-extraction framework.

Information Sources

We searched PubMed, Cumulative Index to Nursing and Allied Health Literature (CINAHL), and Ichushi-Web. Eligible studies were limited to peer-reviewed articles published from 1990 onward. The final database search was conducted on December 25, 2025. Language handling was predefined by the database: English-language search terms were used in PubMed and CINAHL, and both Japanese and English search terms were used in Ichushi-Web.

Search Approach

Searches were conducted primarily in titles and abstracts (where database functions allowed), using combinations of terms for negative interpersonal behavior and outcome domains. Exposure-related terms included, for example, incivility, workplace incivility, rude/rudeness, disrespect, bullying, mobbing, negative acts, harassment, abusive supervision, interpersonal mistreatment, and scale-related terms (e.g., Negative Acts Questionnaire/Negative Acts Questionnaire-Revised (NAQ/NAQ-R), Work Incivility Scale). Outcome-related terms included mental health and work-related indicators such as psychological distress, depression, anxiety, stress, burnout, well-being, job satisfaction, turnover intention, intention to leave, and work-family conflict. For language handling, PubMed and CINAHL searches used English-language terms, whereas Ichushi-Web searches combined Japanese equivalents of the core exposure and outcome terms with English terms to improve the retrieval of relevant Japanese-language studies.

Eligibility Criteria

We included original quantitative studies that met all of the following criteria: participants were university faculty members or higher-education staff; workplace incivility, workplace bullying/mobbing, or workplace cyberbullying was explicitly defined and measured; outcomes included mental health indicators (e.g., psychological distress, burnout, post-traumatic stress disorder (PTSD)-related symptoms, depressive symptoms/disorders) and/or work-related outcomes (e.g., job satisfaction, turnover intention, work engagement, work-family conflict); and exposure-outcome associations were reported quantitatively.

Because the review question focused on university faculty, studies of mixed occupational samples were considered only when university faculty were included and the study provided relevant evidence on negative interpersonal behavior and mental health/work-related outcomes; such studies were interpreted cautiously with respect to generalizability.

Study Selection and Data Extraction

A total of 77 records were screened after duplicate removal. Records were identified from database searches (n = 39) and additional sources (reference-list screening of relevant review articles; n = 43), followed by de-duplication. After title/abstract screening, 17 full-text articles were assessed, and six studies were included in the final review.

Eleven full-text articles were excluded for the following main reasons: no quantitative examination of the exposure-outcome association (n = 7), primary outcomes outside the scope of this review (n = 1), unclear definition of incivility/bullying exposure (n = 2), and full text unavailable (n = 1).

To improve transparency in this single-reviewer process, we used predefined eligibility criteria and a fixed extraction form (author, title, setting/country, participant characteristics, study design, exposure concept/measure, outcome measure, and principal findings), and we re-checked extraction consistency before final synthesis.

For validated instruments used in the included studies, original and/or validation references were provided in the manuscript text, and, as applicable to each study (e.g., instruments for incivility, bullying/mobbing, work stress, cyberbullying, and self-compassion).

Synthesis Strategy

We synthesized findings narratively and organized results by exposure type: workplace incivility, workplace bullying or mobbing, and workplace cyberbullying. We also highlighted mechanistic evidence (e.g., rumination and organizational support) that may explain the pathways from exposure to outcomes.

Findings

Study Selection and Overview

Seventy-seven records were screened, and six studies met the inclusion criteria. The studies were conducted in the United States (four studies), Turkey (one study), and Pakistan and Finland (one study). The study designs included cross-sectional surveys (three studies), a two-wave survey (one study), structural modeling across two studies (one article reporting two studies), and a retrospective record-based clinical sample evaluated for forensic reporting (one study). Table 1 summarizes the characteristics, measures, and results of the included studies.

**Table 1:** Summary of included studies on workplace incivility and bullying and associated outcomes among university faculty Table 1 presents the characteristics and main findings of the included studies. Scale references: Validated instruments used in the included studies are identified in the corresponding table cells and are supported by the original or foundational sources, as applicable, including the Workplace Incivility/Civility Survey (WICS; Clark, 2014; psychometric evidence in Clark et al., 2021), the Workplace Incivility Scale (WIS; Cortina et al., 2001), the Direct and Indirect Aggression Scales-Adult (DIAS-Adult; Österman & Björkqvist, 2009), the Work Stress Symptoms Scale (WSSS; Björkqvist & Österman, 1992), the Survey of Perceived Organizational Support (SPOS/POS; Eisenberger et al., 1986), the Workplace Cyberbullying Measure (WCM; Farley et al., 2016), the Self-Compassion Scale-Short Form (SCS-SF; Raes et al., 2011), the Posttraumatic Stress Diagnostic Scale (PDS; Foa et al., 1997), and the Impact of Event Scale-Revised (IES-R; Weiss & Marmar, 2007), as applicable [2,17-25].

Study (Author/Title)	Country	Participants/Design	Exposure (Concept/Measure)	Outcomes (Measure)	Key Findings
Baran Tatar & Yüksel / Mobbing at workplace: psychological trauma and documentation of psychiatric symptoms (2019) [11]	Turkey	Retrospective, record-based clinical sample (including evaluations for forensic reporting). Among 300 cases evaluated for alleged “workplace trauma” during 2008-2012, 130 cases met criteria for mobbing.	Mobbing (Leymann criteria: ≥1/week for ≥6 months; identified via clinical interview based on five activity domains).	PTSD: PDS (Posttraumatic Stress Diagnostic Scale), IES-R (Impact of Event Scale-Revised); depression and other diagnoses: clinical diagnosis based on Diagnostic and Statistical Manual of Mental Disorders, Fourth Edition, Text Revision (DSM-IV-TR).	PTSD diagnoses were common among mobbing cases (approximately 70% by DSM-IV-TR). Comorbid major depressive disorder was frequent, with many cases receiving multiple diagnoses. IES-R total scores were high.
McGee / The relationship among faculty-to-faculty incivility and job satisfaction or intent to leave in nursing programs in the United States (2023) [12]	United States	Faculty in nursing education programs. Cross-sectional survey (initially planned as stratified cluster sampling but changed to convenience sampling because of low response rates).	Faculty-to-faculty incivility: Workplace Incivility/Civility Survey.	Job satisfaction; intent to leave (additional self-administered items within the same survey).	Higher faculty-to-faculty incivility was associated with lower job satisfaction and poorer retention indicators (higher intent to leave). Approximately half of the participants perceived incivility as a moderate-to-severe problem, and a subset reported low confidence in responding.
Malik & Björkqvist / Workplace bullying and occupational stress among university teachers: mediating and moderating factors (2019) [13]	Pakistan/Finland	University teachers (N = 610; Pakistan n = 329, Finland n = 281). Online cross-sectional survey. Conditional process modeling (PROCESS) was used to examine mediation and moderation.	Workplace bullying: DIAS-Adult subscale (Österman & Björkqvist).	Occupational stress symptoms: Work Stress Symptoms Scale (Björkqvist & Österman). Mediators: colleague and family relationships (study-developed).	Bullying significantly predicted stress symptoms. Family relationships functioned as a mediator (buffering pathway), whereas colleague relationships did not. No moderation by sex or country was observed.
He, Walker, Payne, Miner / Explaining the negative impact of workplace incivility on work and non-work outcomes: the roles of negative rumination and organizational support (2021) [14]	United States	University faculty (N = 154), two-wave survey.	Workplace incivility: Workplace Incivility Scale (Cortina et al.); mediator: negative work rumination; moderators: perceived organizational support and family supportive work environment perceptions.	Work outcomes: job satisfaction, job burnout; non-work outcomes: work-to-family conflict and life satisfaction.	Negative work rumination mediated associations between incivility and both work and non-work outcomes. Perceived organizational support and related contextual factors conditioned the strength of this mediation (moderated mediation).
Miner, Eischeid, Smittick, He, Costa / Organizations behaving badly: antecedents and consequences of uncivil workplace environments (2019) [15]	United States	Two studies using structural equation modeling (unit/department characteristics → uncivil workplace environment → personal incivility experiences → work- and health-related outcomes).	Organizational factors → uncivil workplace environment (WUE) → personal incivility.	Job satisfaction; turnover intentions; psychological distress; physical health.	The model supported pathways in which WUE increased personal incivility experiences, which were in turn associated with poorer outcomes, including lower job satisfaction, higher turnover intentions, greater psychological distress, and poorer physical health.
Ramos Salazar, Weiss, Yarbrough, Sell / The effects of COVID-19 risk, gender, and self-compassion on the workplace cyberbullying and job satisfaction of university faculty（2025) [16]	United States	University faculty. Cross-sectional convenience sample. Of 243 responses, the analytic sample was n = 179 after eligibility screening and missing-data handling.	Workplace cyberbullying (WPCB): Farley et al. Workplace Cyberbullying Measure (10 items; frequency).	Job satisfaction: Agho et al. Job Satisfaction Scale (5 items). Mediator: Self-Compassion Scale-Short Form (Raes et al.).	Correlational analyses showed that WPCB was negatively associated with job satisfaction (r ≈ −0.41), whereas self-compassion was positively associated with job satisfaction. Regression analyses indicated that WPCB predicted lower job satisfaction, and self-compassion functioned as a mediator. Gender and perceived COVID-19 severity risk were associated with WPCB.

Workplace Incivility and Related Outcomes

Faculty-to-faculty incivility in nursing programs: One U.S. study focused on faculty-to-faculty incivility in nursing programs. Incivility was measured using the Workplace Incivility/Civility Survey, and outcomes, including job satisfaction and intent to leave, were assessed using additional survey items. The study reported a negative association between faculty-to-faculty incivility and job satisfaction as well as retention-related indicators. Approximately half of the participants perceived incivility as a moderate-to-severe problem in their workplace. The study also suggested that limited confidence in addressing incivility and fear of retaliation may serve as barriers to corrective action [12].

Negative work rumination and organizational support as pathways: A two-wave survey study of U.S. university faculty examined the pathways linking workplace incivility to work- and non-work-related outcomes. Workplace incivility was measured using the Workplace Incivility Scale, and negative work rumination was measured at the second time point. Outcomes included job satisfaction, burnout, work-to-family conflict, and life satisfaction. This study found that negative work rumination mediated the association between incivility and outcomes, suggesting that uncivil experiences may persist cognitively and influence well-being beyond the immediate event. In moderated mediation analyses, perceived organizational support conditioned parts of the indirect pathway, indicating that organizational support may buffer the impact of incivility by weakening rumination-to-outcome links or altering the strength of the mediation effect [14].

Uncivil workplace environment and organizational antecedents: One article reported two studies examining an “uncivil workplace environment” construct and its consequences. The proposed model linked unit-level organizational characteristics to an uncivil workplace environment, which in turn increased personal experiences of incivility among faculty members and was associated with outcomes including job satisfaction, turnover intentions, psychological distress, and physical health. This approach frames incivility not only as a set of individual acts but also as an emergent workplace climate that may be shaped by organizational features and influence individual exposure and outcomes [15].

Workplace Bullying, Mobbing, and Mental Health-Related Outcomes

Bullying and occupational stress among university teachers: A cross-national survey of university teachers from Pakistan and Finland examined workplace bullying and occupational stress symptoms. Workplace bullying was measured using a subscale from the Direct and Indirect Aggression Scales for Adults (DIAS-Adult), and occupational stress symptoms were assessed using the Work Stress Symptoms Scale. The study used conditional process modeling to examine whether relationships with colleagues and family mediated the association between bullying and stress symptoms, while sex and country served as moderators. Workplace bullying predicted stress symptoms, and family relationships served as a mediator (buffer), whereas relationships with colleagues did not mediate this association. Neither sex nor country significantly moderated this effect [13].

Mobbing and psychiatric symptom documentation in a clinical sample: A Turkish retrospective, record-based study examined individuals referred for psychiatric evaluations related to workplace trauma, including forensic reporting. Among cases presenting with workplace trauma claims, a substantial proportion met criteria for mobbing based on Leymann’s definition (hostile and unethical communication occurring at least weekly for at least six months). Outcomes were assessed through clinical diagnoses based on Diagnostic and Statistical Manual of Mental Disorders, Fourth Edition, Text Revision (DSM-IV-TR) criteria and standardized measures, including the Posttraumatic Stress Diagnostic Scale (PDS) and the Impact of Event Scale-Revised (IES-R). The study documented high rates of PTSD among mobbing cases and frequent comorbidity with major depressive disorder, with many individuals receiving multiple diagnoses. Because this was a highly selected clinical/forensic referral sample, these findings should be interpreted as illustrating the potential upper bound of psychiatric burden under chronic mobbing rather than the typical severity or prevalence of outcomes in general university faculty populations [11].

Workplace cyberbullying and job satisfaction: One U.S. cross-sectional study examined workplace cyberbullying among university faculty members during the COVID-19 pandemic. Workplace cyberbullying was measured using the Workplace Cyberbullying Measure, and job satisfaction was assessed using the Agho et al. Job Satisfaction Scale. The study reported a moderate negative correlation between workplace cyberbullying and job satisfaction and a positive association between self-compassion and job satisfaction. Regression analyses suggested that self-compassion may mediate the association between workplace cyberbullying and job satisfaction. The study also reported associations between workplace cyberbullying and gender and perceived COVID-19 risk, suggesting that both individual and contextual factors may influence exposure during periods of heightened online communication [16].

Interpretation

Principal Findings

This narrative review identified six quantitative studies addressing workplace incivility, workplace bullying/mobbing, and workplace cyberbullying among university faculty. Despite the small number of studies and variability in design and measurement, several consistent themes were identified.

Workplace incivility among faculty members was associated with adverse work-related outcomes. Across studies, greater exposure to incivility has been associated with lower job satisfaction and higher turnover intentions. This pattern is practically important because faculty retention affects educational capacity, mentoring, and institutional continuity, and shortages in health-related disciplines can influence the training pipeline.

Second, cognitive and organizational mechanisms appear to be relevant. In a two-wave study, negative work rumination mediated the association between incivility and work outcomes (job satisfaction and burnout) and non-work outcomes (work-to-family conflict and life satisfaction). This supports a pathway in which low-intensity disrespect persists cognitively and contributes to spillover effects beyond the workplace [14]. Daily diary evidence also suggests a within-person association between incivility and work-related rumination [26].

Third, bullying and mobbing may have more severe mental health consequences than incivility. A faculty bullying study linked bullying to occupational stress symptoms [13], and a clinical/forensic evaluation study documented substantial trauma-related and depressive morbidity among mobbing cases [11]. Importantly, the forensic/clinical mobbing evidence reflects a highly selected, high-severity referral context and should not be used to infer prevalence or typical symptom severity in university faculty populations overall. Rather, it should be interpreted as illustrating the potential upper bound of psychiatric burden under chronic mobbing. Bullying differs from incivility in terms of repetition, persistence, and power imbalance, which may increase feelings of helplessness and perceived threat. The presence of clinically significant trauma-related outcomes suggests that higher education institutions should not treat bullying merely as an interpersonal disagreement.

Fourth, workplace cyberbullying is an emerging concern with limited evidence. The available literature suggests that workplace cyberbullying is negatively associated with job satisfaction and that self-compassion may be a relevant psychological resource. Online interactions can reduce social cues, increase misinterpretation, and enable repeated exposure outside traditional work hours, potentially heightening rumination and spillover effects. However, more studies are required, including studies that account for organizational policies, digital communication norms, and the evolving nature of online work.

Implications for Prevention and Support

This supports the use of preventive approaches at multiple levels. Access to confidential consultations, peer support systems, and training in early responses to low-intensity disrespect may help prevent escalation. The fear of retaliation observed in faculty samples suggests that skill training alone is insufficient if reporting and response systems are perceived as unsafe.

At the organizational level, policies and response systems should address both incivility and bullying. Because incivility can be dismissed as “minor” and targets may fear retaliation, institutions need clear behavioral expectations, protected reporting routes, and credible follow-up processes. For bullying and mobbing, independent channels (e.g., ombudspersons) and access to occupational and mental health services are particularly important. Creating conditions in which faculty can raise concerns without fear of negative consequences, often discussed as psychological safety, may be a practical prerequisite for early consultation and reporting [27].

Regarding bullying and mobbing, institutions should consider stronger protective measures and pathways for providing professional support. Because bullying involves a power imbalance, targets may have limited ability to address the situation through direct confrontation. Independent reporting channels, ombudsperson systems, and access to occupational and mental health services may be important. Documentation practices may also matter, as shown in the forensic evaluation context, although most faculty cases do not involve clinical/forensic assessment.

Health-related faculty may require particular attention because of their heavy workloads, clinical teaching responsibilities, and frequent interactions with multiple professional groups. Faculty well-being and retention have downstream implications for student learning environments and, ultimately, for the healthcare workforce.

Conceptual and Measurement Issues

Interpretation of the current evidence requires attention to conceptual distinctions and operational thresholds. Incivility is typically measured using self-report scales that assess the frequency of low-intensity behaviors. Bullying and mobbing definitions often require sustained exposure (e.g., weekly for six months) and emphasize power imbalance. Studies may differ in whether they capture episodic, chronic, or climate perceptions. Such differences can influence the observed associations with outcomes and the severity of the implications [1-7].

Outcome measures also varied, ranging from self-reported job satisfaction and stress symptoms to clinical diagnoses and standardized trauma measures. Although self-reported outcomes are appropriate for many research questions, combining them with additional sources (e.g., absenteeism, turnover records, or clinical assessments when indicated) could improve validity and clarify severity thresholds relevant for intervention.

Most of the included studies were cross-sectional, limiting causal inference and raising concerns about reverse causation and common-method bias. Longitudinal designs and intervention studies are needed to clarify temporal ordering and evaluate prevention and response strategies [6,7].

Limitations

This narrative review is not intended to be exhaustive. Although we described our search and selection approach, we did not register a protocol or conduct a formal risk-of-bias appraisal. The synthesis was descriptive and did not provide pooled effect estimates. Additionally, the evidence base was small and heterogeneous, limiting the specificity of the recommendations. Therefore, conclusions should be interpreted as an overview of current evidence and key issues rather than definitive effect sizes.

## Conclusions

Among university faculty, available quantitative studies suggest that workplace incivility is associated with poorer work-related outcomes, such as lower job satisfaction and higher turnover intentions, and that negative work rumination may transmit effects to both work and non-work domains. Evidence of bullying and mobbing indicates associations with occupational stress symptoms and, in some settings, a clinically significant psychiatric symptom burden. Evidence for workplace cyberbullying is limited but points to a negative association with job satisfaction and a potential protective role for self-compassion.
